# Reactive Thrombocytosis Related Cerebral Venous Thrombosis: A Rare Complication of Untreated Iron Deficiency Anemia

**DOI:** 10.7759/cureus.19064

**Published:** 2021-10-26

**Authors:** Shashi Maryala, Aparna Vaddiparti

**Affiliations:** 1 Neurology, George Washington University, Washington, USA; 2 Neurology, Gandhi Hospital, Hyderabad, IND; 3 Neurology, University of Connecticut, Hartford Hospital, Hartford, USA; 4 Neurology, University of Alabama at Birmingham School of Medicine, Birmingham, USA

**Keywords:** polycystic ovary syndrome (pcos), secondary/reactive thrombocytosis, reactive thrombocytosis, severe iron deficiency anemia, cerebral venous sinus thrombosis (cvst)

## Abstract

Thrombocytosis can be either primary or secondary, and it can cause venous pro-thrombotic states like cerebral venous thrombosis. Untreated iron deficiency anemia is postulated to cause reactive (secondary) thrombocytosis due to the proliferation of common progenitor cells. Here we present a case of a middle-aged woman with polycystic ovary syndrome and episodes of menorrhagia. She presented with headache and focal sensory deficits, and her neuroimaging showed evidence of cerebral venous sinus thrombosis (CVST). Laboratory tests showed microcytic hypochromic anemia, low ferritin, high total iron-binding capacity (TIBC), and thrombocytosis with a platelet count of 1,523,000/mm³. A comprehensive workup for hypercoagulable states and primary causes of thrombocytosis was negative. It was concluded that the etiology of her CVST was a reactive thrombocytosis from chronic untreated iron deficiency anemia. Anticoagulation with apixaban and corrective treatment for iron deficiency anemia was initiated. A repeated neuroimaging after four months showed significantly less clot burden in the cerebral venous sinuses, and then apixaban was stopped after six months. Laboratory tests after one year of iron replacement therapy showed improvement in the hemoglobin and hematocrit as well as normalization of platelet count. This case highlights a rare yet potentially dangerous complication of a common untreated condition, i.e., iron deficiency anemia.

## Introduction

Iron deficiency anemia is commonly seen in women of childbearing age [1]. Chronic untreated iron deficiency anemia is postulated to cause reactive (secondary) thrombocytosis due to the proliferation of common progenitor cells. Here we present a unique case of a reactive thrombocytosis resulting in cerebral venous thrombosis. 

## Case presentation

**Figure 1:**
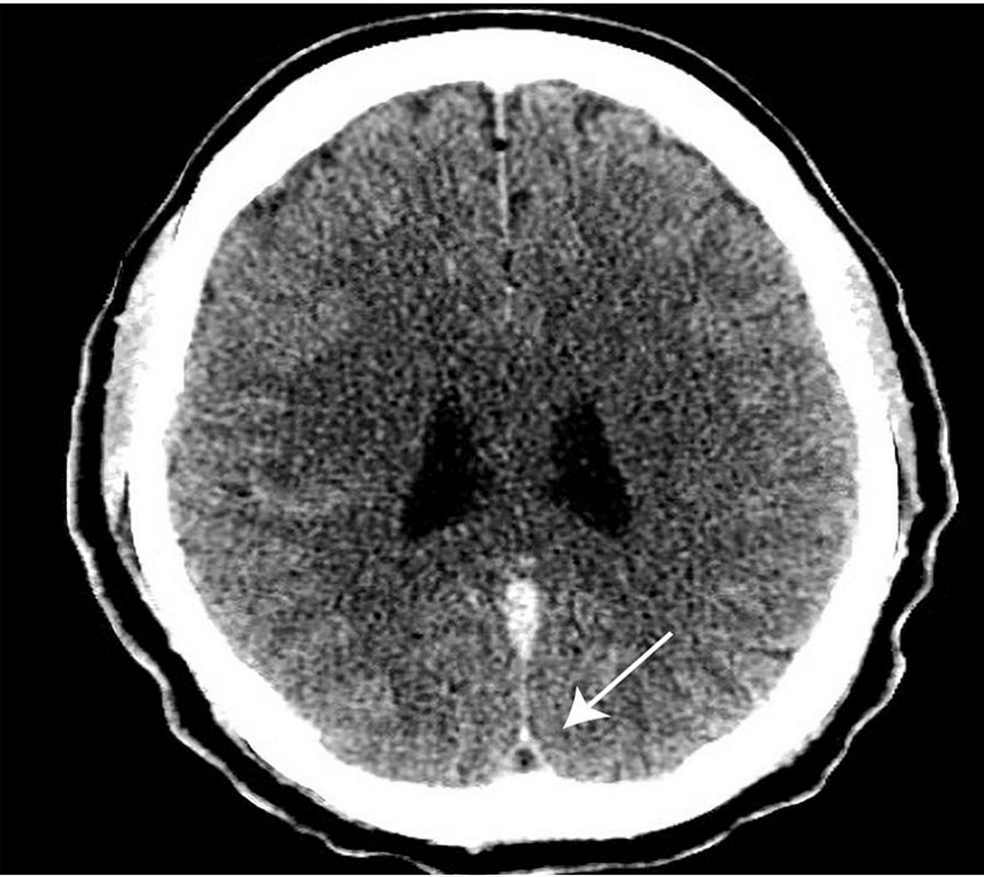
CT angiogram (axial sequence) showing "empty delta" sign.

A 41-year-old African American woman presented with headaches and numbness in the left face and upper limb. Her medical history was notable for hypertension and polycystic ovary syndrome (PCOS) with episodes of menorrhagia. A neurological exam showed subtle hypoesthesia to pinprick in the left face and upper arm. Labs showed severe microcytic, hypochromic anemia with a hemoglobin level of 6.4 g/dl, a hematocrit value of 25.3%, and platelets at 1,523,000/mm³. Low serum ferritin and a high total iron-binding capacity (TIBC) were noted consistent with the pattern of iron deficiency anemia. CT venography (Figure 1) showed a filling defect in the superior sagittal sinus (SSS) extending to the confluence of sinuses, referred to as the “empty delta" sign.

**Figure 2:**
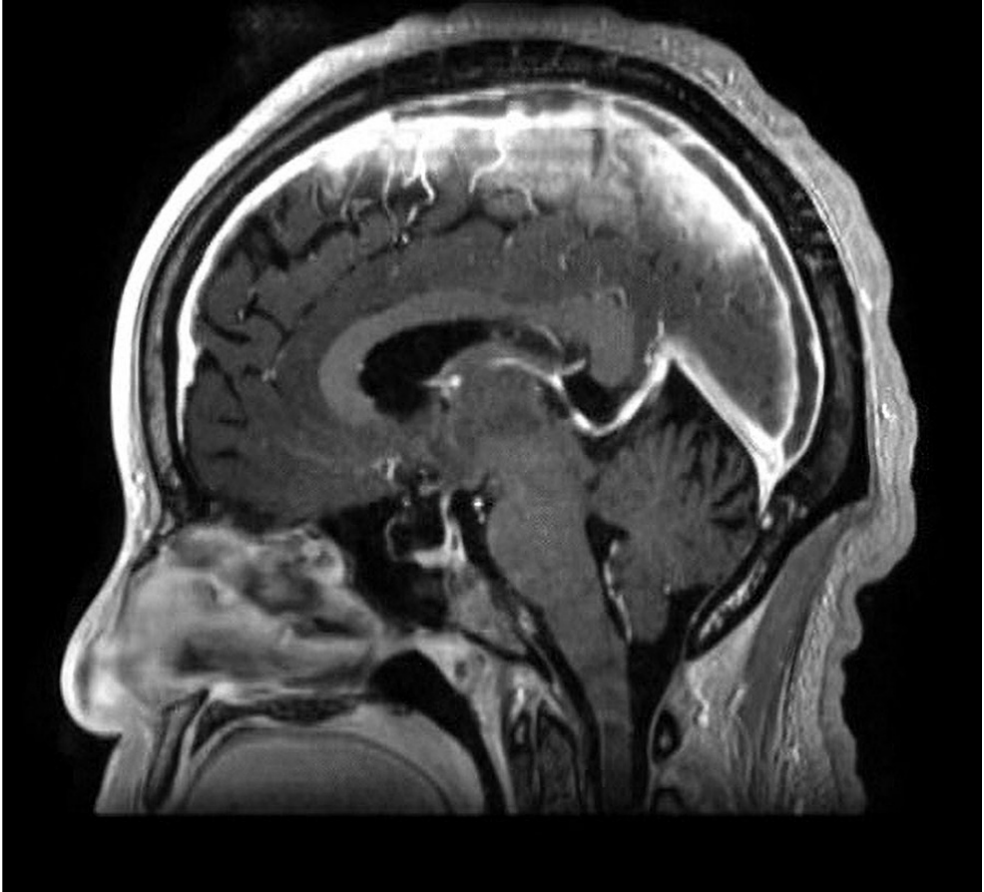
MR cerebral venography (MRV) showing cerebral venous sinus thrombosis (CVST) in the superior sagittal sinus extending into the confluence of sinuses.

MR cerebral venography (Figure 2) confirmed the presence of cerebral venous sinus thrombosis (CVST) in the superior sagittal sinus (SSS) and the confluence of sinuses extending anteriorly into the right frontal cortical vein.

**Figure 3:**
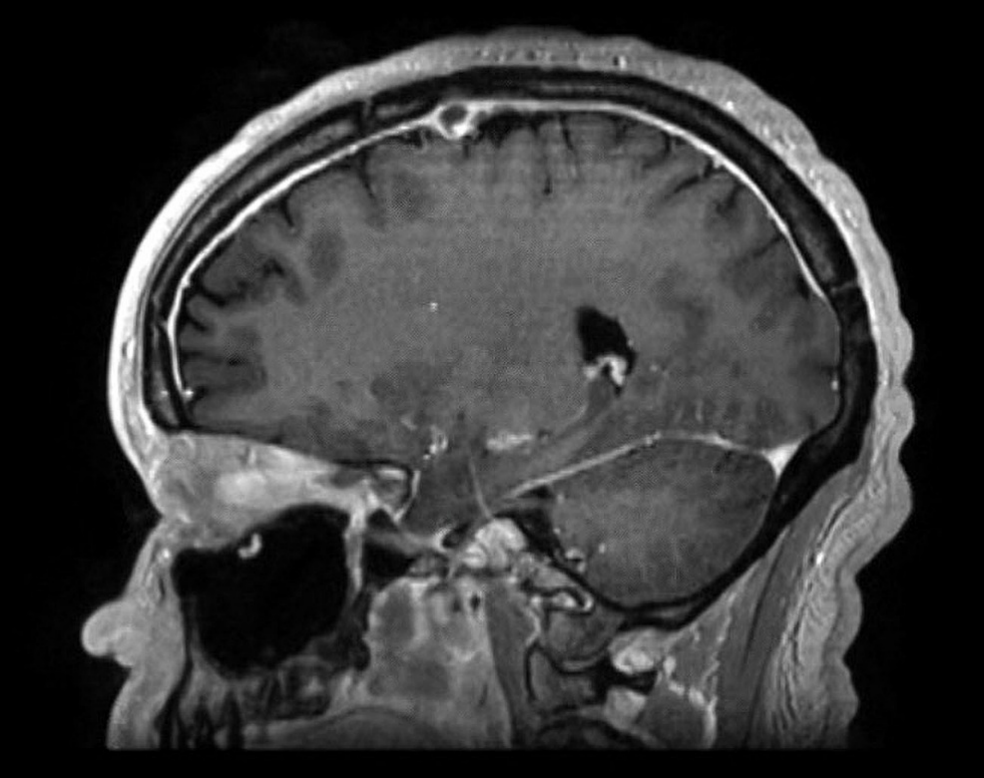
MRI brain: post-contrast sagittal sequence showing pachymeningeal enhancement.

Post-contrast images on MRI (Figure 3) showed pachymeningeal enhancement related to venous congestion without any signs of acute ischemia or hemorrhage.

The patient was immediately started on a low molecular weight heparin infusion. An extensive workup for potential etiologies of the CVST, especially those contributing to thrombocytosis, was undertaken. Work-up, including JAK2 kinase mutation for primary thrombocytosis, bone marrow biopsy and BCR-ABL mutation for myeloproliferative disorders, hemoglobin electrophoresis to detect the presence of HbS or HbC, and a comprehensive screen for hypercoagulable states, including Factor V Leiden mutation, prothrombin mutation, and antithrombin III deficiency, were all negative. Based on the clinical history and extensive investigations, it was concluded that her CVST was likely related to the reactive (secondary) thrombocytosis from the chronic untreated iron deficiency anemia. 

Treatment and follow-up

Corrective treatment for iron deficiency anemia was initiated, and heparin was transitioned to apixaban. A repeat MRI of the brain performed at four months post-discharge showed resolution of the pachymeningeal enhancement, and MR venography showed significant improvement in the clot burden in the cerebral venous sinuses. A complete blood picture (CBC) at one-year follow-up showed improvement in hemoglobin levels to 10.1 g/dl and hematocrit to 33.2%. There was also normalization of the platelet count to 445,000/mm³. Apixaban was stopped after six months, and corrective treatment for the iron-deficiency anemia was continued along with hematology follow-up. 

## Discussion

Thrombocytosis, defined as platelet count > 450,000/mm³, can be (A) primary from genetic causes like JAK2 kinase mutation and myeloproliferative disorders like chronic myeloid leukemia; or (B) secondary from acute infections, chronic inflammatory states, iron deficiency anemia, post-splenectomy, and malignancies [2]. While primary thrombocytosis is known to cause thromboembolic complications, the clinical significance of reactive thrombocytosis is less recognized [3]. The rate of thrombocytosis in patients with iron deficiency anemia was reported to be as high as 33% [4], with a strong negative correlation with the severity of anemia [5,6]. Untreated iron deficiency anemia, which causes elevated levels of erythropoietin, is postulated to cause increased platelets in addition to red blood cells (RBCs) due to the proliferation of common progenitor cells in the bone marrow [7]. A similar sequence of amino acids between erythropoietin and thrombopoietin, the predecessors of red blood cells and platelets, respectively, has been proposed as a potential mechanism by which elevated erythropoietin may lead to thrombocytosis [8].

In our case, a patient with chronic undiagnosed, and thus untreated, iron deficiency anemia and resultant reactive thrombocytosis led to the development of cerebral venous sinus thrombosis. Other factors potentially contributing to prothrombotic state in our patient include transferrin upregulation due to iron deficiency anemia [9] as well as reduced fibrinolysis and increased plasminogen activator inhibitor (PAI-1) associated with PCOS [10,11] and/or its metabolic sequelae such as insulin resistance and obesity [12]. The timely initiation of iron replacement therapy along with anti-coagulation for the CVST showed improvement in the hematological profile with both correction of anemia and normalization of platelet count and demonstrated notable clot burden reduction in the cerebral venous sinuses.

## Conclusions

Thrombocytosis, even if reactive/secondary, need to be recognized as a risk factor for thromboembolic states such as cerebral venous thrombosis. It is imperative to conduct extensive work-up for both primary and secondary causes of thrombocytosis.

Our case highlights a potentially dangerous complication-CVST, of a very common condition-iron deficiency anemia; it also underscores the value of timely treatment given the reversible nature of both the underlying condition and its complication.
